# Promoting Household Water Treatment through Women's Self Help Groups in Rural India: Assessing Impact on Drinking Water Quality and Equity

**DOI:** 10.1371/journal.pone.0044068

**Published:** 2012-09-05

**Authors:** Matthew C. Freeman, Victoria Trinies, Sophie Boisson, Gregory Mak, Thomas Clasen

**Affiliations:** 1 Department of Environmental Health, Center for Global Safe Water, Rollins School of Public Health, Emory University, Atlanta, Georgia, United States of America; 2 Faculty of Infectious Disease, Environmental Health Group, London School of Hygiene and Tropical Medicine, London, United Kingdom; 3 Department of Environmental Health, Mailman School of Public Health, Columbia University, New York, New York, United States of America; U. S. Salinity Lab, United States of America

## Abstract

Household water treatment, including boiling, chlorination and filtration, has been shown effective in improving drinking water quality and preventing diarrheal disease among vulnerable populations. We used a case-control study design to evaluate the extent to which the commercial promotion of household water filters through microfinance institutions to women's self-help group (SHG) members improved access to safe drinking water. This pilot program achieved a 9.8% adoption rate among women targeted for adoption. Data from surveys and assays of fecal contamination (thermotolerant coliforms, TTC) of drinking water samples (source and household) were analyzed from 281 filter adopters and 247 non-adopters exposed to the program; 251 non-SHG members were also surveyed. While adopters were more likely than non-adopters to have children under 5 years, they were also more educated, less poor, more likely to have access to improved water supplies, and more likely to have previously used a water filter. Adopters had lower levels of fecal contamination of household drinking water than non-adopters, even among those non-adopters who treated their water by boiling or using traditional ceramic filters. Nevertheless, one-third of water samples from adopter households exceeded 100 TTC/100ml (high risk), and more than a quarter of the filters had no stored treated water available when visited by an investigator, raising concerns about correct, consistent use. In addition, the poorest adopters were less likely to see improvements in their water quality. Comparisons of SHG and non-SHG members suggest similar demographic characteristics, indicating SHG members are an appropriate target group for this promotion campaign. However, in order to increase the potential for health gains, future programs will need to increase uptake, particularly among the poorest households who are most susceptible to disease morbidity and mortality, and focus on strategies to improve the correct, consistent and sustained use of these water treatment products.

## Introduction

Unsafe drinking water is a leading cause of preventable disease, particularly among the young, the immuno-compromised and the poor [1]. Diarrhea represents a significant share of this burden, causing an estimated 4 billion cases and 1.9 million deaths each year of children under 5 years, or 19% of all such deaths in developing countries [2]. Because they lack safe water and sanitation, low-income populations bear much of this disease burden [3]. Lower levels of education by caregivers and distance to improved water supplies are also risk factors for diarrhea in children. With over 386,000 deaths attributable to diarrheal diseases per year, India ranks first among countries contributing to this worldwide disease burden [4].

India has made considerable progress in recent years in improving water supplies in both rural and urban settings [5]. However, only 12% of the rural population is served by a household connection [6]. Moreover, surveys of microbial water quality throughout India have shown extensive fecal contamination of drinking water supplies. In Hyderabad, for example, 50% of water samples drawn from pre-monsoon, monsoon, and post-monsoon period were positive for fecal coliforms [7]. In Madhya Pradesh, 33% of boreholes were fecally contaminated [8]. Even water that is safe at the point of distribution is subject to frequent and substantial contamination during collection, transport, and storage [9].

Household water treatment and safe storage (HWTS), including boiling, chlorinating, and filtering water at home, offers the potential for addressing both uncertain water quality at the point of delivery and post-collection contamination. Systematic reviews of water quality interventions have shown HWTS – including filtration and chlorination – can be effective in improving the quality of drinking water and in preventing diarrhea [10]–[12]. Studies have also shown more traditional HWTS practices – such as boiling – can also be microbiologically effective [13], [14]. Based on this evidence, the World Health Organization (WHO) and UNICEF recommend HWTS for populations relying on unsafe water supplies as part of a comprehensive strategy to prevent diarrheal disease, particularly among young children [4].

Nevertheless, the potential of HWTS has not been realized at scale, largely because of the challenge of reaching the most vulnerable populations with effective HWTS options that are accessible, acceptable and affordable and that they will use correctly and consistently on a sustainable basis [15]. While boiling is the most common method of treating water at home worldwide and has been shown to be effective in India [13], only about 10% of the Indian population reports boiling their water before drinking it [16]. Chlorinating water with sodium hypochlorite, an intervention that has achieved scale elsewhere, has not been successfully promoted in India. Gravity water filters, including traditional ceramic “candle” filters and, more recently, a variety of commercial filters that incorporate disinfection media, are one HWTS option that has achieved scale nationally. However, the up-front cost of microbiological quality filters has generally made them prohibitively expensive for low-income populations who are at greatest risk [15].

Promotion through microfinance institutions (MFIs) may offer a way to overcome the barriers of access to and affordability of HWTS. A study of a school-based promotion approach in India revealed the potential for micro loans offered by women's self-help groups (SHGs) to provide needed financing to reach the poorest households [17]. SHGs have also been shown to provide a critical social network that may lead to increased uptake among marginalized populations [18], [19]. In 2007, Hindustan Unilever Limited (HUL) and ACCESS Development Services (ACCESS), a support organization for an alliance of microfinance institutions, began a pilot program to explore the potential for using microfinance institutions to improve awareness of and access to a commercial HWTS product among lower-income rural populations in India. Through the program, SHG members were offered the opportunity to purchase HUL's Pureit® filter and micro loans to mitigate the up-front cost. Researchers undertook this study to evaluate the extent to which the pilot program improved drinking water quality among the target population, a condition to achieving health gains. If proven effective, this mechanism seems plausible for scale-up for this and other HWTS solutions given high MFI penetration and participation throughout India.

## Methods

### Ethical considerations

The study was reviewed and approved by the Ethics Committee of the London School of Hygiene and Tropical Medicine and the Institutional Review Board (IRB) of Columbia University Mailman School of Public Health. The study was exempt from ethics approval in India because it falls under the provisions of program evaluation [20]. An information sheet providing complete details of the study, advising that participation was entirely voluntary, and assuring participants of the confidentiality of their personal data was read to all participants and written consent was recorded. Respondents did not receive compensation for their participation.

### Program background

HUL, a subsidiary of Unilever Ltd., is one of the largest consumer products companies in India. ACCESS, originally organized by CARE, an international non-governmental organization (NGO), provides organizational and technical assistance to an alliance of over 110 non-profit microfinance institutions (MFIs) in the Indian states of Andhra Pradesh and Tamil Nadu that each support a network of women's self-help groups (SHGs). The objective of the pilot program was to improve the drinking water quality of lower-income households who were not reached through normal commercial channels by increasing their awareness of effective HWTS methods – including boiling, filtration, and chlorine additives – and extending access to HUL's Pureit filter as well as micro loans to purchase a filter.

The Pureit filter removes microbial contaminants through chlorination and carbon filtration; treated water is safely stored in a residual chamber where it is accessed via a tap [21]. The unit retailed for 1,500 Indian Rupees (RS) (US$32) at the start of the pilot program and sold for Rs 2,000 (US$43) at the time of the study. Pureit provides continuous filtration for 1,500 L of drinking water, at which point the unit's end of life meter indicates the need to replace the consumable “battery” (prefilter, chlorine cartridge and carbon media) at a cost of Rs 350 (US$7).

Under the pilot program, HUL representatives gave presentations to SHG members about the sources and risks of contaminated drinking water and methods to effectively treat their water at home, including boiling, filtration, and chlorination, and gave a demonstration of the Pureit filter. After the presentation, SHG members were informed that they could take out a micro loan to purchase the Pureit filter. Repayment terms varied but adopters typically made a down payment and paid the remainder in installments over 8–18 months with 1–2% interest per month. HUL supplied the MFI a commission of 10% off the retail cost. After receiving orders, HUL technicians delivered and set up the filters at the purchaser's house. As of June 2009, eleven ACCESS MFI affiliates covering 262,353 members had agreed to participate in the program. Of these, 67,230 members (25.6%) had received the presentations and 6,556 Pureit filters had been sold (representing 9.8% of households exposed to the program).

### Study design

The study was conducted between September and October 2009 in Andhra Pradesh. The evaluation was conducted as a case-control study following a design developed in part by Khan [22]. A case was defined as a female SHG member whose household had acquired a Pureit; a control was defined as a female member of an SHG that offered loans for Pureit but who had not purchased the product. Community respondents were also interviewed to provide comparison on several metrics. Resources allowed for an expected sample size of 300 cases and 300 controls. Sampling was done through a clustered approach due to logistic considerations such as the lack of centralized records. Villages served as the primary sampling unit and adopters/non-adopters as the secondary sampling unit.

### Sampling strategy

At the time of the study, eight MFIs in Andhra Pradesh had partnered with ACCESS and HUL to offer their members loans for Pureit. For logistical reasons, four were included in the study: PSS in Warangal, CAMP in Guntur, CAMEL in Sullurupet and IWB in Nellore. The number of villages and respondents under each MFI were selected proportionally to the number exposed to the program. Villages were randomly selected until we could identify a sufficient number of adopters. Selected villages were in rural and peri-urban areas. Due to the need for daily testing of water samples, villages more than 1.5 hours from the central water testing facility were not eligible for inclusion. Villages were also excluded when there were insufficient records to gather a list of SHG members and where there was no field staff at either HUL or the MFI available to help the survey team locate respondents. Following these eligibility criteria, a total of 33 villages were surveyed. Respondents were selected from MFI records using systematic random sampling. For the purposes of this paper, we define purchasers of Pureit as “adopters,” and adopters who met criteria for use (treated current drinking water with Pureit) as “users.” Approximately eight systematically selected community members were interviewed in each village in order to assess equity of SHG membership and whether the controls were representative of the broader community.

### Household surveys and water sampling methods

Surveys were conducted in Telugu by a contracted team of five professional surveyors. The female head of household was asked about water handling and treatment practices, Pureit purchase and use history, and demographic information. Household assets and use of water treatment methods was confirmed with observation when possible.

Following administration of the survey, a sample of water was collected directly from the storage vessel the informant identified to be used for drinking. In order to assess how the household sample compared with the water supply (well, tap, etc.) from which it was drawn, samples were also collected from community water sources. Community water sources were identified with the aid of village informants and sampled and coded prior to surveying households. Due to limited analytic capacity, a single source water sample was used for all households that reported collecting water from that source. In places where households reported individual water taps piped from a central source, one sample was taken from a randomly selected household to serve as the source sample of all households using that piped water supply.

Samples were collected in sterilized Whirl-pac® bags containing sodium thiosulfate to neutralize possible residual chlorine to simulate water quality at the time of use. Samples were placed in a cooler for transport and processed within 5 hours of collection. A 100 ml sample was passed through a 0.45 µ membrane filter (Millipore Corporation, Bedford, Massachusetts, USA) and incubated on membrane lauryl sulphate media (Oxoid Limited, Basingstoke, Hampshire, England) at 44°C±0.5°C for 18 hours. The number of yellow colonies were counted and recorded as individual thermotolerant coliforms (TTC), all in accordance with *Standard* *Methods* [23] and the instructions and material from the Oxfam-Delagua portable water testing kit (http://www.delagua.org/). In order to meet WHO guidelines for drinking water quality, 100 ml samples should be free of TTC; samples containing 1–10, 11–100, and 101–1000 cfu/100 ml of TTC are considered low risk, moderate risk, and high risk, respectively [24].

### Data analysis

Data were entered twice into Microsoft Excel 2008 and cross-checked for consistency. Data were cleaned and analyzed in SPSS v.18 (Chicago, IL), SAS v9.2 (Cary, NC) and STATA v.10 (College Station, TX). To assess the equity of adoption of Pureit, we conducted a logistical regression using adoption status as the key dependent variable. Independent covariates included socio-economic status (SES), family size, presence of children under 5 years, education level for male and female heads of household, observed presence of soap, presence of latrine, reported handwashing practices, water source type, and distance to water source. SES was assessed using a wealth score derived from an index of house assets calculated using principal components analysis [25]. To assess equity of membership in an MFI, adopters and non-adopters were compared to non-members; responses were weighted based on the overall adoption status in the study area of 8.8%.

Differences between adoption status and water quality measures were assessed using colony counts as the dependent variable. Samples that exhibited contiguous growth of bacteria or were otherwise too numerous to count (TNTC) were given the value of 1.5 times the highest number of colonies of the samples that were still countable for statistical and analytical purposes. Tests were performed to compare household and source water quality and to compare water quality to adoption status and water treatment practices. Due to over-dispersion of data and excess 0 values, analysis of water quality samples utilized a negative binomial distribution. To compare changes between source and stored drinking water, colony counts were normalized using a log_10_ transformation and the difference between stored and household water samples were calculated. For this purpose, colony counts of zero were assigned values of 1 added in order to allow for log_10_ transformation. The resulting distribution approximated normality and estimates of effect were calculated using student's t-test.

Population level estimates were calculated using the SVY command in STATA. Where source water was included in the analysis, standard errors were adjusted to account for clustering at the water source level. Sampling weights were employed at the MFI level based on probability of selection.

## Results

### Study population and adoption

Our analysis covers 281 adopters, 247 non-adopters and 251 other community members (Table 1). Sixteen adopters (5.7%) were eliminated from analysis because no Pureit filter was observed at their house. A total of 53 (10.0%) surveys were discarded due to incomplete records. During the initial 18 months of the program, 3,651 filters were purchased among 41,290 SHG members exposed to the program in the four MFIs included in the study, an adoption rate of 8.8% (Table 1). Ninety-nine (35.0%) of Pureit users had replaced the battery of consumables at least once (range 1–7). At the time of the investigator's visit, however, the end-of-life indicator on 163 (62.9%) of Pureit devices showed the need to replace the consumable components.

**Table 1:** Sample sizes, program delivery information and adoption rates by microfinance organization.

	Total	PSS	CAMP	CAMEL	IWB
Adopters sampled	281	125	41	80	35
Non-Adopters sampled	248	70	52	87	39
Community members sampled	251	104	24	88	35
Villages sampled	33	14	9	5	5
Exposed Members	41,290	29,169	3,764	5,663	2,694
Filters sold	3,651	2,784	261	425	181
Member Adoption rate	8.8%	9.5%	6.9%	7.5%	6.7%

### Demographics

Table 2 shows key demographic characteristics and water management practices for adopters, non-adopters and community members generally. Adopters were more likely than non-adopters to have children under 5 years old (21.5% vs.13.0%, p<0.01) and had a greater percentage that had at least some secondary education among male (79.5% vs. 56.2%, p = 0.02) and female heads of household (64.9% vs. 39.0%, p = 0.05). Adopters were more likely than non-adopters to be in the highest 20% and next 40% wealth quintiles.

**Table 2:** Household demographics and water treatment practices among adopters, non-adopters, and community members generally.

Variable	Adopters	Non-adopters		Community Members	
	*n = 265*	*n = 247*	*p^1^*	*n = 247*	*p^2^*
Mean age of respondent	35.4 (9.7)	36.4 (9.5)	0.30	34.8 (9.8)	0.04
Mean household size	4.4 (1.4)	4.3 (1.5)	0.25	4.3 (1.7)	0.97
Households with children under 5	57 (21.5%)	32 (13.0%)	0.02	58 (23.2%)	<0.01
Education of male household head					
No formal education	26 (9.9%)	57 (23.1%)	ref	39 (15.7%)	ref
Some/completed primary school	28 (10.6%)	51 (20.7%)	0.80	36 (14.5%)	0.93
At least some secondary school	211 (79.5%)	139 (56.2%)	<0.01	175 (69.8%)	0.03
Education of female household head					
No formal education	44 (16.6%)	90 (36.6%)	ref	73 (29.1%)	ref
Some/completed primary school	49 (18.5%)	60 (24.4%)	0.10	53 (21.1%)	0.80
At least some secondary school	172 (64.9%)	96 (39.0%)	<0.001	125 (49.8%)	0.12
Asset score					
Poorest 40%	56 (22.0%)	126 (52.5%)	ref	111 (46.4%)	ref
Middle 40%	125 (49.0%)	82 (34.2%)	<0.001	87 (36.4%)	0.72
Least poor 20%	74 (29.0%)	32 (13.3%)	<0.001	41 (17.2%)	0.33
Electricity	265 (100.0%)	244 (98.8%)	0.11	246 (98.4%)	0.67
Current water source is improved	238 (89.8%)	229 (92.7%)	0.54	208 (82.9%)	<0.01
Current water source is improved within 1 KM	101 (44.1%)	125 (38.1%)	0.08	85 (33.9%)	<0.01
*Water treatment*					
Reported treating current water for drinking	245 (92.5%)	144 (58.3%)	<0.001	165 (65.7%)	0.47
Treatment method used currently	230 (81.8%)	81 (32.8%)	<0.001	116 (46.2%)	0.01
Pureit	209 (77.4%)	0 (0.0%)	<0.001	7 (2.8%)	0.03
Boiling	13 (4.6%)	21 (8.5%)	0.02	30 (12.0%)	0.06
Ceramic filter	16 (5.7%)	60 (24.3%)	<0.001	83 (33.2%)	0.07
Treatment methods used previously	140 (49.8%)	103 (41.7%)	0.14	143 (57.0%)	<0.01
Boiling	35 (12.5%)	48 (19.4%)	0.03	71 (28.3%)	<0.01
Ceramic filter	114 (40.6%)	66 (26.7%)	0.03	97 (38.7%)	0.07

Data are mean (SD) or count (%) weighted for probability of selection. “*ref” refers to the referent group. p^1^* is the probability of the difference between adopters (cases) and non-adopters (controls) being attributable to chance. *p^2^* is the probability of the difference between MFI members and community members being attributable to chance.

Although similar in socio-economic indicators, community members were different from MFI members in important respects (Table 2). Community members were more likely to have a child under 5 (23.2%, p<0.01), but were slightly better educated. Heads of household were more likely to have some secondary education for males (69.8%, p = 0.03), but not females (49.8%, p = 0.12). The distribution of community members into SES quintiles was similar to that of the MFI members. Community members were more likely than non-adopters to have used microbiologically effective HWT methods, such as boiling, filtration, or Pureit (46.2% vs. 32.8%, p<0.01).

### Water treatment practices

Adopters were more likely to report treating their current drinking water (92.5%) than non-adopters (92.5% vs. 58.3%, p<0.001) (Table 2). A greater percentage of non-adopters reported boiling their water (19.4%) or using ceramic filters (24.3%) than adopters (12.5%, p = 0.02 and 5.7%, p<0.001, respectively). No respondents reported treating their water with chlorine in liquid or tablet form. Adopters were more likely to report relying on ceramic filtration (40.6%) before purchasing Pureit than non-adopters (26.7%) (p = 0.03), but less likely to boil (12.5% vs. 19.4%) (p = 0.03). Combining these suggests that adopters were more likely to use potentially effective HWT methods prior to their acquisition of Pureit (49.8%) than non-adopters (41.7%), but the difference was not statistically significant (p = 0.14). Fewer than 80% of households with Pureit observed in their home reported using Pureit to treat their current water and only 73% were using their unit to store water at the time of the survey.

Approximately half (46.2%) of community members reported treating their water prior to drinking. One third (33.2%) reported using a ceramic water filter, while 12.0% reported boiling. This was higher than members of the MFI (p<0.01 for both). Only 7 (2.8%) of community members had purchased the Pureit filter.

### Water quality

Water quality samples included 520 households and 33 source water samples. A total of 273 adopters (97%) and 247 non-adopters (98%) had complete data for household and source samples and were used for analysis.

In general, water quality at the source was of poor microbiological quality, with a geometric mean coliform count of 144.6 (95%CI: 78.8–210.4) TTC/100 ml (Table 3). Fewer than 10% of source samples met the WHO Guidelines for safe drinking water by being free of detectible TTC; 70% of source water samples exceeded 100 TTC/100 ml (high risk) and another 15% of source water samples had TTC counts between 11–100/100 ml (moderate risk).

**Table 3:** Arithmetic and geometric means of water samples collected from adopters and non-adopters.

	n	Arithmetic mean (95% CI)	Geometric mean (95% CI)	*p*
*Thermotolerant coliform (TTC) counts for household and source water* †				
Household	490	153.2 (134.8; 171.6)	24.8 (19.9; 30.8)	0.75
Source	33	144.6 (78.8; 210.4)	29.9 (13.0; 68.7)	
*Source water quality (TTC) by adoption status* * †				
Adopter	243	212.1 (188.4; 235.8)	65.3 (49.7; 85.8)	0.96
Non-adopter	247	213.4 (191.1; 235.7)	101.7 (82.4; 125.4)	
*Household water quality (TTC) by adoption status* †				
Adopter	243	118.4 (95.4; 141.3)	13.7 (9.9; 18.8)	0.01
Non-adopter	247	187.5 (159.3; 215.7)	44.5 (33.7; 58.8)	
*Household water quality (TTC) by water treatment method* †				
Confirmed Pureit use	186	86.6 (64.3; 108.8)	9.2 (6.5; 13.0)	ref
Other filter methods	72	93.4 (53.9; 133.1)	15.5 (9.3; 25.9)	0.79
Reported boiling	24	209.7 (130.3; 289.1)	55.0 (19.2; 157.0)	0.04
Unsafe or no method	208	227 (194.7; 259.3)	64.4 (47.7; 86.8)	<0.01
*Log reduction in TTC from source to household by adoption status* § *				
Adopter	243	0.64 (0.47; 0.82)		0.24
Non-adopter	247	0.36 (0.21; 0.50)		
*Log reduction in TTC from source to household by water treatment method* § *				
Confirmed Pureit use	186	0.80 (0.61; 1.00)		ref
Other filter methods	72	0.87 (0.58; 1.17)		0.59
Reported boiling	24	0.18 (−0.29; 0.64)		0.04
Unsafe or no method	208	0.13 (−0.01; 0.28)		0.01

*p* values determined by

§ T-tests or

† negative binomial regression.

*“ref” refers to the referent group.*

* *Variance adjusted for clustering at source water sample.*

**Figure 1:**
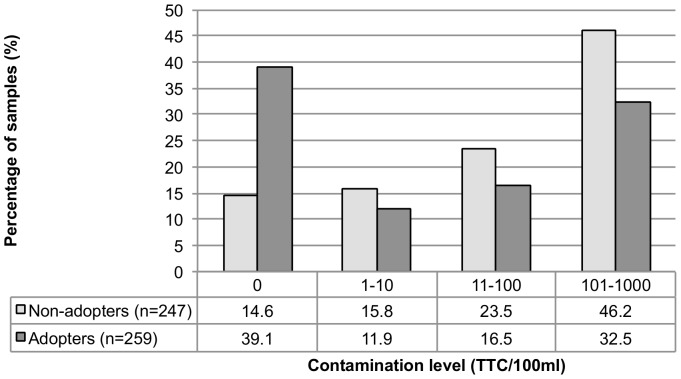
Household water quality among Pureit adopters and non-adopters.

Household samples overall had lower levels of fecal contamination compared to source, with a geometric mean of 24.8 (95%CI: 19.9–30.8) TTC/100 ml (Table 3). Among the overall study population, 39.5% of household samples had contamination levels in excess of 100 TTC/100 ml, and 20.2% were of moderate risk. About one quarter (26.3%) of household samples had no detectible fecal contamination (Figure 1). Pureit users whose device indicated the need for a replacement battery had higher levels of fecal contamination in their drinking water than those with a functioning battery, though that difference was marginally insignificant (p = 0.06) (data not shown).

**Figure 2:**
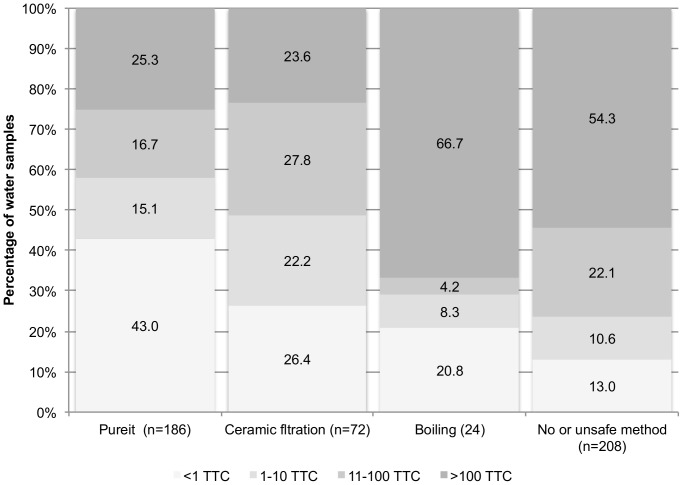
Household water quality of respondents using Pureit, other safe treatment methods or no or unsafe treatment methods.

Among household samples, adopters had better water quality than non-adopters (Table 3). The geometric mean TTC count was 13.7 (95%CI: 9.9–18.8) among adopters, and 44.5 (95%CI: 33.7–58.8) among non-adopters (p<0.01). While 46.2% of non-adopters had contamination in excess of 100 TTC/100 ml, 32.5% of adopters met this high-risk level of contamination. Nearly 40% of adopters had no detectible TTC in their household water, compared to 14.6% among non-adopters, regardless of actual reported treatment practices (p<0.01) (Figure 2).

We further explored water quality by reported and observed current treatment practices (Table 3). The geometric mean TTC count was lower for households using Pureit (9.2; 95%CI: 6.5–13.0) than those that reported boiling (55.0; 95%CI 19.2–157.0; p = 0.04) and those that reported no or no safe treatment (64.4; 95%CI 47.7–86.8; p<0.01); however, it was not significantly different than those who used a ceramic filter (15.5; 95%CI: 9.3–25.9) (p = 0.79). TTC count was highest for those using unsafe methods such as cloth filtration or no method (64.4; 95%CI: 47.7–86.8). Respondents that treated their current water with Pureit had the highest proportion of their household samples (43.0%) free of fecal contamination compared to respondents who treated with boiling (p<0.01), those who used a ceramic filter (p = 0.01), or those that used unsafe methods or used no method (p<0.001) (Figure 2). Pureit users had similar percentage of samples with TTC counts in excess of 100/100 ml (25.3%) compared to those that used ceramic filters (23.6%) (p = 0.78); these values were different than boilers (p<0.001) and those that reported no safe method (p<0.001).

Table 3 also compares the log_10_ difference between source and household water quality by reported treatment method. Adopters and non-adopters had no difference in the change in water quality from source to home. There was a difference between Pureit users and boilers (p = 0.04) and those who used no or no safe method (p = 0.01). However, there was no significant difference between Pureit users and those who reported using ceramic filters (p = 0.59).

### Equity and water quality

**Figure 3:**
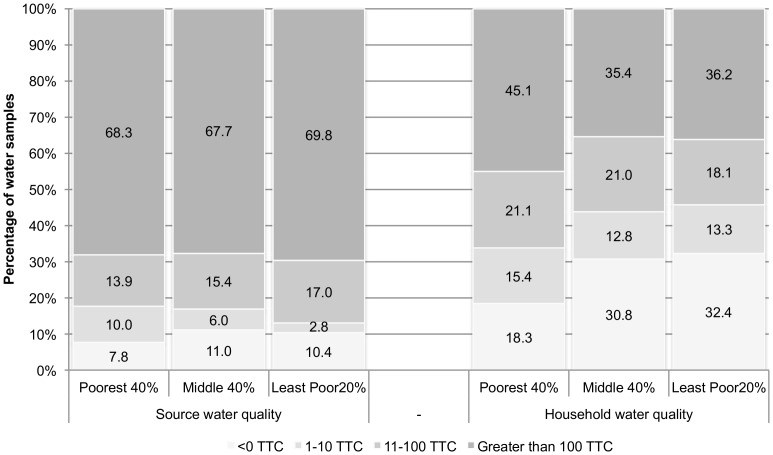
Source and household water quality by wealth tercile.

We assessed the water quality for sampled source and household water by a number of key equity variables independent of intervention status. While source water quality was similar between SES quintiles, household water quality varied (Figure 3). The poorest households had a smaller proportion of samples with no detectable TTC/100 ml as compared to the least poor (18.3% vs. 32.4%, p = 0.007), and a greater proportion of samples with more than 100 TTC/100 ml in their household water (45.1% vs. 36.2%, p = 0.14), though the difference was not statistically significant.

A significance test of the log reduction of contamination between source and household water quality, regardless of adoption status, revealed that the difference between the least poor and poorest socio-economic tercile was significant at p<0.1 (data not shown). When regressed against the linear combination of wealth and Pureit adoption, we found that wealth tercile was a significant effect modifier in the improvement between source and household water quality (p = 0.04 if tercile 2 vs. 1, though p = 0.63 for tercile 3 vs. 1). Given positive indication for effect modification, we present separate estimates of effect for each SES tercile (Table 4). These data suggests that poorer households were less likely to achieve improvement in water quality from using Pureit than adopters from higher economic strata. Adopters in the poorest 40% of the population showed no significant improvement in water quality (β −0.11, p = 0.66). However, those in the middle 40% had a 0.56 log improvement in water quality (p = 0.05); among the least poor 20%, adopters had a commensurate improvement in water quality (β 0.49, p = 0.12).

**Table 4:** The log improvement of water quality (TTC/100 ml) between household and source water modeled against adopters status and Pureit use, for each socio-economic tercile.

	Pureit adopters vs. non adopters			Pureit use vs. non use		
Variable	β	CI	*p value*	β	CI	*p value*
*Model 1: unadjusted*	0.49	0.6; 0.92	0.03	0.28	0.25; 0.82	0.29
*Model 2: SES*						
Poorest 40%	−0.11	−0.62; 0.40	0.66	0.11	−0.40; 0.61	0.64
Middle 40%	0.56	0.004; 1.12	0.05	0.62	−0.04; 1.27	0.06
Least poor 20%	0.49	−.13; 1.11	0.12	0.41	−0.26; 1.08	0.22

*β are log improvements in water quality between the source and household point-of-use calculated using linear regression of log-transformed values.*

## Discussion

Overall, the pilot program resulted in increased water quality among households that had adopted a Pureit water filter; however, the program had only limited success in achieving its aim of providing safe drinking water to a vulnerable population. This was in part due to the tendency of adopters to come from potentially lower risk strata; they had better socioeconomic indicators and were more likely to have previously used effective HWTS products, in this case boiling and ceramic water filters. In addition, there were sub-optimal improvements in water quality even among adopters, likely due to lack of correct and consistent use of the device.

During the initial 18-month period of the pilot program, 9.8% of SHG participants exposed to the program acquired Pureit filters. On the one hand, this is an impressive level of market penetration for a consumer durable in a low-income, rural population sold through commercial channels, particularly during the early stage of a product's life cycle. Even socially marketed health products rarely achieve this level of penetration [15]. Moreover, any level of sales where the cost is borne by the beneficiary reduces the outlay that the public sector or civil society must make in order to achieve higher levels of coverage. Additional efforts are underway in India promote water filters to SHG members using microfinance [26]. It is possible that these efforts will benefit from additional experience and achieve higher levels of penetration in the target population. Ultimately, however, the potential contribution of HWTS will depend on the extent to which it achieves scale. The relatively low levels of uptake when promotion is conducted through commercial channels has led promoters of other household-based health products, such as insecticide treated mosquito nets for preventing malaria, to abandon a commercial strategy in favor of free distribution [15].

These results provide some evidence that the program was not optimizing improved access to safe drinking water for segments of the population that may be most vulnerable. Half of current Pureit users reported a history of boiling or using ceramic filters, a higher percentage of safe water practice than among non-adopters. This indicates that a disproportionate number of households were simply substituting the new HWTS technology for another water treatment method shown to reduce pathogen contamination. Pureit users did have better water quality than those boiling, and Pureit has been shown to be capable of significantly higher levels of microbiological performance than ceramic filters, particularly in respect of viruses [11]. However, we did not observe a statistically significant difference in the log reduction value achieved by Pureit compared to ceramic filters in this study. Although adopters were more likely to have children <5, who are at greatest risk of mortality from unsafe drinking water [27], non-adopters were more likely to fall into vulnerable demographic categories, including lower education, less wealth, and poorer access to an improved water source within 1 KM. In addition, Pureit adopters in the poorest economic quintile did not achieve the same improvement in water quality as those in higher quintiles. Promotion through SHGs may need to include greater targeting of the most vulnerable households so that the program moves beyond increasing the number of products available on the market to enabling health behavior change.

Despite starting with comparable source water quality, Pureit adopters had better drinking water quality at the household level on average than non-adopters, even though non-adopters included households that reported boiling their water or using ceramic filters. However, a substantial proportion of adopters continued to be exposed to high levels of fecal contamination in the water they reported drinking. These results are comparable to the improvement in water quality in a study of self-reported boilers in India [13]. They demonstrate that possessing the hardware for effectively treating water at home is not sufficient for ensuring safe drinking water at the household level.

One possible explanation for the sub-optimal household water quality among adopters of Pureit is the apparent low rates of compliance (correct, consistent use). Consistent use in areas with high pathogen contamination has been shown to be more critical than slight differences in pathogen reduction [28]. Only 73% of households observed to have a Pureit were using their unit to store water at the time of the survey, and fewer than 80% of adopter households reported using Pureit to treat the water they were actually drinking. Over two-thirds of Pureit users required a replacement battery and yet many were continuing to use the filter. There was some evidence that water quality was poorer among those whose battery needed replacement [29]. HUL reports that it has since modified the Pureit to block the water flow and thus render it unusable when the disinfection agent has been exhausted. However, the apparent issues concerning sustained use of the filter may arise chiefly from limited awareness of need, access and affordability of the consumables, a challenge that is not addressed with this hardware fix. Consistent and sustained use of household water treatment methods is a frequently discussed challenge and indicates that any future promotional programs should have a greater focus on behavior change [15], [30].

This study offers mixed evidence on the merits of enlisting MFIs and SHGs in drinking water initiatives. There were no significant differences between the SES profile of SHG members and community members, and rates of using effective water treatment methods – filters or boiling – were similar if not higher among non-SHG community members. However, community members were more likely to have children under 5 and less likely to have access to an improved water source. While focusing on SHG members may miss some vulnerable households, there are indications that such programs do reach a relatively equitable cross-section of the population.

There were a number of key limitations to this study. First, it was challenging to develop a comprehensive sampling framework. Not all microfinance organizations keep diligent records of members who purchased Pureit or even membership records. We constructed the most comprehensive list of adopters and non-adopters with the available information. However, sampling bias may impact our findings. Second, our water sampling methods were limited by available resources and the inability to run a suitable number of dilutions. Of the 506 household water samples collected, 60 (11.9%) had coliform counts that were too numerous to count (TNTC) and 5 of 33 (15.2%) of source water samples were found to be TNTC. We followed standard protocols for working with lab results truncated by detection limits, but the potential for bias remains. Third, the clustered nature of the data and the analytical requirements of using these type of data limited our power to reject the null hypothesis regarding the equity of water quality measures.

When assessing the success of a program to improve water quality to improve health, it is common to rely on the number of units in use or calculate the percentage uptake of a particular product as evidence of delivery of safe water. The implication is that adoption of a particular method confers benefits of the water treatment technology revealed from effectiveness trial data. However, our findings point to the need for better monitoring approaches to ensure that the technology is meeting those in greatest need. Indicators to consider include previous water handling practices, socio-economic indicators and water quality improvements in order to fully understand who is benefitting from a distribution program. The challenge of sustained correct use of the product echoes similar challenges of HWTS discussed elsewhere [12], [15].
